# Multilayer Connector Hub Mapping Reveals Key Brain Regions Supporting Expressive Language

**DOI:** 10.1089/brain.2020.0776

**Published:** 2021-02-12

**Authors:** Brady J. Williamson, Manlio De Domenico, Darren S. Kadis

**Affiliations:** ^1^ Department of Radiology, University of Cincinnati, Cincinnati, Ohio, USA.; ^2^ Fondazione Bruno Kessler, Center for Information and Communication Technology, Trento, Italy.; ^3^ Neurosciences and Mental Health, Research Institute, Hospital for Sick Children, Toronto, Ontario, Canada.; ^4^ Department of Physiology, University of Toronto, Toronto, Ontario, Canada.

**Keywords:** connector hubs, expressive language, multilayer networks, multimodal imaging

## Abstract

***Introduction:*** How components of the distributed brain networks that support cognition participate in typical functioning remains a largely unanswered question. An important subgroup of regions in the larger network are *connector hubs*, which are areas that are highly connected to several other functionally specialized sets of regions, and are likely important for sensorimotor integration. The present study attempts to characterize *connector hubs* involved in typical expressive language functioning using a data-driven, multimodal, full multilayer magnetoencephalography (MEG) connectivity-based pipeline.

***Methods:*** Twelve adolescents, 16–18 years of age (five males), participated in this study. Participants underwent MEG scanning during a verb generation task. MEG and structural connectivity were calculated at the whole-brain level. Amplitude/amplitude coupling (AAC) was used to compute functional connections both within and between discrete frequency bins. AAC values were then multiplied by a binary structural connectivity matrix, and then entered into full multilayer network analysis. Initially, hubs were defined based on multilayer versatility and subsequently reranked by a novel measure called delta centrality on interconnectedness (DCI). DCI is defined as the percent change in interfrequency interconnectedness after removal of a hub.

***Results:*** We resolved regions that are important for between-frequency communication among other areas during expressive language, with several potential theoretical and clinical applications that can be generalized to other cognitive domains.

***Conclusion:*** Our multilayer, data-driven framework captures nonlinear connections that span across scales that are often missed in conventional analyses. The present study suggests that crucial hubs may be conduits for interfrequency communication between action and perception systems that are crucial for typical functioning.

**Impact statement:**

We present methodology to characterize regions supporting cross-frequency communication in the distributed language network. There are 3 key innovations: (1) incorporation of a structural connectivity constraint based on diffusion magnetic resonance imaging (MRI), (2) use of a full multilayer framework that captures both within- and between-frequency connections, and (3) introduction of a new metric, delta centrality on interconnectedness (DCI), that quantifies the importance of a region for cross-frequency coupling.

## Introduction

Current models of how the brain supports expressive language function are often based on conventional, task-based, active-baseline contrast neuroimaging analyses that fail to differentiate activity that is necessary for completing a task (i.e., task-essential) versus activity that is task-correlated, but not necessarily crucial for completion. Recently, there has been a paradigmatic shift in neuroimaging from active-baseline subtraction (conventional approach) to connectivity-based analyses. Importantly, decreased activation in a region, measured by functional magnetic resonance imaging (fMRI), may be associated with increased connectivity to other regions (Büchel et al., 1999; Kelly and Garavan, 2004; McIntosh et al., 1999). Regions that may be increasingly important in the network as a conduit of connectivity are potentially de-emphasized in activation-based studies. Network analysis provides a framework in which to study the global and local topological features of brain networks (Rubinov and Sporns, 2010), which may be more informative about which regions are crucial for a task.

Brain connectivity can be represented as an adjacency matrix, consisting of brain regions (i.e., nodes) and their connections (i.e., edges). Within this network, centrality metrics can be used to determine which nodes are most important (i.e., network *hubs*) (van den Heuvel and Sporns, 2013b). Hubs can be further split into two types: *connector hubs,* which are highly connected to several different functionally specialized sets of regions, and *provincial hubs,* which are highly connected within one functionally specialized set of regions (Rubinov and Sporns, 2010; van den Heuvel and Sporns, 2013a). Connector hubs, due to their common placement at the interface between sensory and motor systems, may be of particular importance for tasks that rely heavily on integration between sensory and motor information, such as expressive language (Pulvermüller, 2018). Defining connector hubs may provide more specificity in characterizing both location and functionality of areas involved in expressive language.

We have previously shown that connectivity-based hub analysis of broadband magnetoencephalography (MEG) and fMRI data can successfully delineate regions thought to be critical for language in children and adolescents performing auditory verb generation (Youssofzadeh et al., 2017, 2018). However, we have also shown that patterns of connectivity differ across the spectra (Kadis et al., 2016); broadband approaches may obfuscate important frequency-specific information. Multilayer network analysis is a mathematical framework that can be used to model and analyze multivariate and multiscale data (De Domenico, 2017; De Domenico et al., 2013; Kivelä et al., 2014). This approach has been used successfully to identify hubs that can separate healthy individuals from clinical populations, to provide evidence that brain function is nontrivially constrained by brain architecture, and to show that models of the brain that allow regions to operate and couple at multiple frequencies better predict empirical MEG data (Battiston et al., 2017; Brookes et al., 2016; De Domenico et al., 2015b, 2016; Deco et al., 2017; Tewarie et al., 2016).

The present study aims to expand on our previous findings by demarcating connector hubs and utilizing unique information from multiple frequency bins. Structural connectivity, derived from diffusion tractography, can be used to inform functional connectivity to restrict connections to only those that are biologically plausible. The current study utilizes generalized Q-sampling imaging (GQI) for reconstruction of diffusion imaging data, on which deterministic tractography is performed (Yeh et al, 2013, 2010). This method remains sensitive to crossing fibers, which occurs in up to 90% of white matter, while limiting false positives (Jeurissen et al., 2013).

The current study seeks to define connector hubs that are important for successful execution of expressive language in typically developing adolescents. We hope to demonstrate that reranking nodes defined as hubs by multilayer versatility according to a novel metric sensitive to a node's importance in interconnectivity of the network will lead to more precise maps of regions thought to be crucially involved in language functioning. Connector hubs will be defined by using a data-driven, structurally constrained, MEG connectivity-based multilayer framework. This framework can be extended to study any cognitive domain and has several real-world applications. Clinically, this could potentially lead to more accurate mapping of eloquent tissue, needed in presurgical planning for patients undergoing cortical resection. Information derived from this pipeline could be used to inform computational models to better replicate brain function and its relation to behavior. Testable generative models of the brain are needed to move beyond description and toward new predictions and theories (Betzel and Bassett, 2017).

## Methods

### Institutional review board approval

This study involving human subjects research was approved by the Institutional Review Board (IRB) at Cincinnati Children's Hospital Medical Center and was carried out in accordance with the ethical standards of title 45, part 46, and title 21 parts 50 and 56, of the Code of Federal Regulations.

### Informed consent

All subjects provided written informed consent or parental consent and children assent in accordance with the Declaration of Helsinki.

### Participants

The study cohort consisted of 15 typically developing adolescents, ages 16–18 years. Inclusion criteria were being a native English speaker without history of neurological insult or disease, speech or language disorder, or learning disability. All participants underwent neuropsychological assessment, MRI, and MEG. Three participants were excluded due to issues of data quality, leaving 12 participants for the final analysis (*M =* 16.89 ± 0.67). The Edinburgh Handedness Inventory (Oldfield, 1971) indicated that all participants were right handed (*M* = 94.68 ± 8.44).

### MRI acquisition

Three-dimensional-T1-weighted (TR/TE = 8.055/3.68 ms, 1.0 × 1.0 × 1.0 mm voxels) and diffusion (*b* = 800 s/mm^2^, 32 directions, 1 b0, TR/TE = 8955/77 ms, 1.875 × 1.875 × 2.37 mm voxels, 55 slices) scans were collected for each participant. Multimodal radiographic markers were placed before the MRI at nasion and periauricular points to facilitate registration with MEG.

### MEG acquisition

MEG data were collected on a 275-channel CTF system (MEG International Services Ltd., Coquitlam, BC, Canada) with a sampling rate of 1200 Hz. A covert verb generation task was used. Nouns and speech-shaped noise were auditorily presented to participants (71 nouns, 72 noise). Participants were instructed to think of a corresponding verb when they heard a noun, and provide no response when they heard noise. Stimuli were presented every 5 sec by an MEG-compatible, calibrated audio system that comprised distal transducers, tubing, and ear inserts (Etymotic Research, IL) and randomly alternated between conditions. During the MEG session, head localization coils were placed at nasion and periauricular points to monitor movement and facilitate coregistration between MEG and MRI.

### MRI processing

Diffusion processing included geometric distortion correction, eddy current correction, Gibbs ringing removal, denoising, and registration of the diffusion and structural images. To improve geometric distortion correction and registration, an “imitation” T2-weighted image was constructed from each participant's T1-weighted image to better match the contrast of the b0 image. The imitation T2 is simply the T1 image (dark gray matter, bright white matter, and dark cerebrospinal fluid [CSF]), with the contrast adjusted to match that of a T2 image (bright gray matter, dark white matter, and bright CSF). See Supplementary Figure S1 for examples of images at each MRI processing step. Structural and diffusion preprocessing was carried out in AFNI and TORTOISE, respectively (Cox, 1996; Irfanoglu et al., 2018; Pierpaoli et al., 2010).

Diffusion processing included geometric distortion correction, eddy current correction, Gibbs ringing removal, denoising, and registration of the diffusion and structural images. To improve geometric distortion correction and registration, an “imitation” T2-weighted image was constructed from each participant's T1-weighted image to better match the contrast of the b0 image. Geometric distortion correction involved aligning the b0 image to the imitation T2, then correcting distortions using nonuniform B-spline grid sampling (Irfanoglu et al., 2011). Gradient vectors were rotated according to the eddy correction and registration. Before reconstruction, the quality of each data set was assessed by calculating the correlation between neighboring diffusion directions/volumes. If more than 10% of the data were excluded due to poor correlation (*r* < 0.9), the data set was excluded. All data were visually inspected after preprocessing to ensure adequate alignment.

Spin distribution functions for each voxel were obtained using GQI (Yeh et al., 2010). GQI was chosen due to the sensitivity to crossing fibers and because it is one of the few higher order diffusion models that can be applied to any diffusion sampling scheme that is balanced, that is, the isotropic voxels are reconstructed as an isotropic spin distribution function (SDF), which is checked during reconstruction (Yeh et al., 2010). Q-space diffeomorphic reconstruction (QSDR), an extension of GQI, involves aligning the subject's quantitative anisotropy map (QA; derived from SDFs) to a QA template in Montreal Neurological Institute space (HCP-842) using diffeomorphic mapping to allow both linear and nonlinear alignment, applying the inverse Jacobian to the subject space SDFs, and adjusting for scaling differences between the subject and template, conserving the amount of diffusion spins in the original data (Yeh and Tseng, 2011). Alignment quality was assessed by the correlation between subject and template SDFs. Default QSDR settings were used except for the number of fibers resolved, which was reduced to three due to the limited directions in the current data set.

Deterministic tractography (curvature limit = 45°, min length threshold = 30 mm, max length threshold = 300 mm, one round of topology-informed tract trimming) (Yeh et al., 2018), based on QA, was performed between cortical parcels. The QA threshold was automatically determined by 0.6*Otsu's threshold, which has been shown to be optimal for resolving true connections while limiting false positives (Maier-Hein et al., 2017; Otsu, 1979). Resulting adjacency matrices were binarized. Diffusion reconstruction and tracking were performed in DSI Studio.

### MEG processing

A bandpass filter from 0.1 to 100 Hz was applied to continuous MEG data. A sharp discrete Fourier transform filter was applied at 60 Hz to reject line noise. Data were initially epoched to −400 to 2000 ms relative to stimulus onset. Jump artifacts were removed based on deviation from a median filter. Participants were excluded if more than 10% of trials were rejected during preprocessing. The period of −400 to 0 ms was used for baseline correction and 0 to 2000 ms was used to capture dynamics related to the task. MEG and MRI data were coregistered using the fiducial markers and single shell head models were constructed from the segmented MRI (Nolte, 2003).

The covariance matrix used for source estimation was constructed from the longer trial epoch (−400 to 2000 ms). Centroids of each parcel in the Brainnetome atlas, consisting of 246 regions (123 per hemisphere) (Fan et al., 2016), were used in calculation of the leadfield matrix, and the time-series at each position was estimated using a linearly constrained minimum variance beamformer (Van Veen et al., 1997) with 0.1% regularization. Noise estimates for each “virtual sensor” were also projected and used to normalize each estimated time course (i.e., compute the neural activity index). The linearly constrained minimum variance natively produces estimates of activity in the three canonical planes. For this study, we project along the dominant orientation, which is equivalent to taking the first eigenvector of the time series.

### MEG connectivity analysis

Source activity was symmetrically orthogonalized to reduce signal leakage (Colclough et al., 2015) and bandpass filtered into equally spaced 5 Hz bins from 0.5 to 50.5 Hz. The resulting frequency-specific time series were cropped to 800–1300 ms relative to stimulus onset. Previous studies using the same task with *visual* stimuli revealed a strong beta-event-related desynchrony around 300–800 ms after stimulus onset as a signature for verb generation (Kadis et al., 2011). The current time window is shifted to account for delay differences between visual and auditory stimulus delivery and perception, as supported by time/frequency analysis (Youssofzadeh et al., 2017). Amplitude envelopes were obtained using a Hilbert transform. Amplitude/amplitude coupling (AAC) was calculated for each node (centroid) pair both within- and between-frequency bins, from low to high frequencies only (Brookes et al., 2012). AAC was chosen as the connectivity metric because it can be applied both within and between frequencies and has been used previously in MEG multilayer network analyses (Brookes et al., 2016; Mandke et al., 2017; O'Neill et al., 2015; Tewarie et al., 2016).

### Normalization with surrogates

Surrogate time-series were calculated to determine which connections were statistically significant. One hundred null models were generated for each source using the 0 to 2000 ms time window, using iterative amplitude-adjusted Fourier transform (Schreiber and Schmitz, 1996). Data were cropped to 800–1300 ms, and null connectivity was computed for each set of surrogates. After obtaining the mean and deviation of the surrogates, z-scores were obtained for empirical connections. This approach resulted in 10 adjacency matrices weighted by z-score (4 within frequency, 6 between frequency). All MEG processing was performed with the Fieldtrip toolbox (Oostenveld et al., 2011) in MATLAB 2016a (The MathWorks, Inc., Natick, MA), and using custom MATLAB routines.

### Multilayer network analysis

**FIG. 1.**
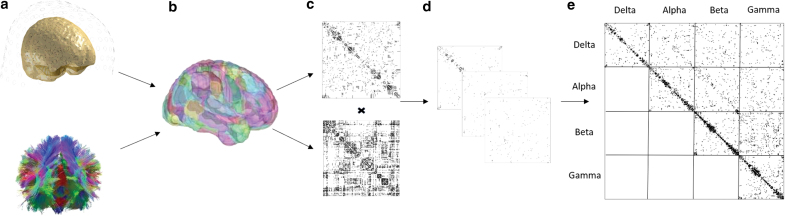
Key steps involved in multilayer analyses. **(a)** Source time-series estimates are obtained from MEG data and tractography performed for diffusion data for each subject, **(b)** connectivity is computed between nodes for each modality, **(c)** weighted connectivity matrices are resolved; the structural matrix (diffusion) is binarized and used to constrain the functional (MEG) matrices by entry-wise multiplication, **(d)** all structurally constrained matrices (both within and between frequencies) are combined into a multilayer network, and **(e)** network measures are computed on the supra-adjacency matrix. While this study used equally spaced bins, the figure shows an example using four canonical bins for simplicity. MEG, magnetoencephalography. Color images are available online.

Adjacency matrices from the MEG connectivity analysis were thresholded using the orthogonal minimum spanning tree method (Dimitriadis et al., 2017). Supplementary Figure S2 shows increased specificity of results in regions most linked to expressive language when using OMST versus a percentage thresholding (i.e., top 10% of connections). Each thresholded MEG connectivity matrix was then multiplied entry-wise by the binary structural connectivity matrix. In matrices characterizing interfrequency connections, the maximum connectivity value was taken between each pair of regions to ensure that the off-diagonal matrices were symmetric and undirected. They were combined to form a full multilayer network, represented by a supra-adjacency matrix (De Domenico et al., 2015b). For further details about the calculation of multilayer versatility, see Appendix A1. Researchers have shown that calculating centrality measure in a single layer or aggregating over multiple layers may be misleading compared with a full multilayer representation. One of the key reasons for this is that single-layer analyses do not capture nonlinear relationships between nodes. Simulations have shown the importance of nonlinear connectivity afforded by cross-frequency connections (Deco et al., 2017). Supplementary Figure S3 shows the difference in using a multilayer framework with and without interconnections. Inclusion of interconnections (full multilayer) also increases the specificity in regions specific to expressive language. Multilayer networks were constructed with in-house scripts and the MuxViz package (De Domenico et al, 2015a) in R (R Core Team, 2018). The pipeline as described to this point is shown in Figure 1.

### Characterizing connector hubs

**FIG. 2.**
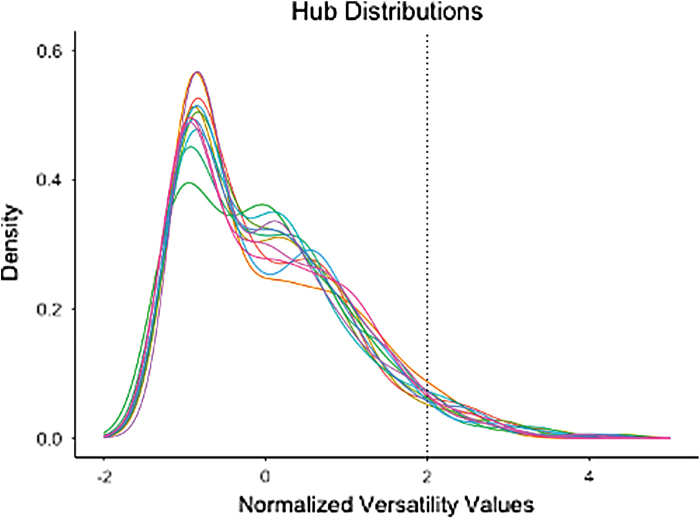
Normalized centrality distribution for all participants. Each color corresponds to a different subject. Dotted line (*x* = 2) is the threshold that was used to determine whether a node was hub based on its multilayer versatility. The plot shows that this threshold successfully captures the expected fat tail of the centrality distribution for all subjects, which is indicative of hubs within the network. Color images are available online.

To determine which nodes were hubs, multilayer versatility (multilayer adaptation of PageRank centrality) was calculated for all nodes (De Domenico et al., 2015b). Hubs were defined as nodes with a z-score-normalized multilayer versatility >2. Human brain networks have been shown to have positively skewed degree distribution, resembling an exponentially truncated power-law distribution (Achard et al., 2006). Although the distribution varies for each participant, this threshold captures the extreme positive values for all participants (Fig. 2). Nodes with suprathreshold-normalized multilayer versatility were then passed to connector hub analysis.

The current study attempts to use a more direct measure of connector hubs. Different brain regions are thought to communicate at multiple frequencies (Deco et al., 2017). It seems feasible that connector hubs may facilitate communication among these multiple frequencies between modules. One measure that may capture this property is interconnectedness (Baggio et al., 2016). More interlayer connections mean the network has higher interconnectedness. For this study, interconnectedness was simply defined as the number of interlayer connections.

To capture local properties of the network, interconnectedness was used to calculate delta centrality for each node. Delta centrality is the percent change in a global network measure after the removal of a node (Fornito et al., 2016). Each hub was iteratively removed, and the percent change in interconnectedness was calculated. The new metric, called delta centrality on interconnectedness (DCI), was used to rerank the original hubs. Since most DCI values for each participant were 0, DCI was centered and scaled around 0. Fisher's one-sample, one-tailed permutation testing was used to determine which hubs had a DCI significantly >0 across all participants, with bootstrapping (5000 iterations) performed to determine significance. Connector hubs in this framework are defined as the regions that significantly increase the overall interfrequency connectedness of the network.

## Results

### Multilayer matrix sparsity

Mean sparsity for each individual layer is reported in Table 1. Independent two-sample *t*-tests revealed no significant differences in sparsity across layers between males and females (corrected *p* > 0.05).

**Table 1.** Mean and Standard Deviation of Sparsity Across Layers for Whole Cohort and for Males and Females, Separately

Mean sparsity for each frequency bin (%)			
Layer (Hz)	Overall mean sparsity	Males	Females
0.5–5.5 to 0.5–5.5	0.030 ± 0.052	0.039 ± 0.069	0.023 ± 0.040
0.5–5.5 to 5.5–10.5	0.080 ± 0.066	0.116 ± 0.064	0.055 ± 0.058
0.5–5.5 to 10.5–15.5	0.058 ± 0.064	0.063 ± 0.077	0.055 ± 0.058
0.5–5.5 to 15.5–20.5	0.067 ± 0.064	0.087 ± 0.075	0.053 ± 0.056
0.5–5.5 to 20.5–25.5	0.048 ± 0.060	0.084 ± 0.071	0.022 ± 0.037
0.5–5.5 to 25.5–30.5	0.066 ± 0.073	0.103 ± 0.087	0.039 ± 0.053
0.5–5.5 to 30.5–35.5	0.084 ± 0.070	0.126 ± 0.069	0.054 ± 0.057
0.5–5.5 to 35.5–40.5	0.062 ± 0.068	0.093 ± 0.079	0.040 ± 0.054
0.5–5.5 to 40.5–45.5	0.063 ± 0.057	0.055 ± 0.064	0.068 ± 0.057
0.5–5.5 to 45.5–50.5	0.036 ± 0.051	0.031 ± 0.051	0.040 ± 0.054
5.5–10.5 to 5.5–10.5	0.008 ± 0.000	0.008 ± 0.000	0.008 ± 0.000
5.5–10.5 to 10.5–15.5	0.080 ± 0.066	0.116 ± 0.064	0.055 ± 0.058
5.5–10.5 to 15.5–20.5	0.058 ± 0.064	0.063 ± 0.077	0.055 ± 0.059
5.5–10.5 to 20.5–25.5	0.067 ± 0.064	0.087 ± 0.075	0.053 ± 0.056
5.5–10.5 to 25.5–30.5	0.059 ± 0.065	0.087 ± 0.075	0.040 ± 0.054
5.5–10.5 to 30.5–35.5	0.083 ± 0.070	0.126 ± 0.069	0.053 ± 0.056
5.5–10.5 to 35.5–40.5	0.057 ± 0.061	0.061 ± 0.073	0.054 ± 0.057
5.5–10.5 to 40.5–45.5	0.092 ± 0.065	0.119 ± 0.066	0.072 ± 0.060
5.5–10.5 to 45.5–50.5	0.092 ± 0.065	0.116 ± 0.064	0.075 ± 0.065
10.5–15.5 to 10.5–15.5	0.008 ± 0.000	0.008 ± 0.000	0.008 ± 0.000
10.5–15.5 to 15.5–20.5	0.062 ± 0.069	0.073 ± 0.089	0.054 ± 0.058
10.5–15.5 to 20.5–25.5	0.084 ± 0.070	0.073 ± 0.089	0.092 ± 0.059
10.5–15.5 to 25.5–30.5	0.079 ± 0.065	0.093 ± 0.079	0.069 ± 0.057
10.5–15.5 to 30.5–35.5	0.031 ± 0.055	0.063 ± 0.077	0.008 ± 0.000
10.5–15.5 to 35.5–40.5	0.058 ± 0.062	0.084 ± 0.071	0.039 ± 0.052
10.5–15.5 to 40.5–45.5	0.094 ± 0.067	0.149 ± 0.024	0.055 ± 0.059
10.5–15.5 to 45.5–50.5	0.112 ± 0.053	0.149 ± 0.024	0.085 ± 0.052
15.5–20.5 to 15.5–20.5	0.008 ± 0.000	0.008 ± 0.000	0.008 ± 0.000
15.5–20.5 to 20.5–25.5	0.054 ± 0.057	0.054 ± 0.063	0.054 ± 0.057
15.5–20.5 to 25.5–30.5	0.090 ± 0.064	0.097 ± 0.083	0.086 ± 0.054
15.5–20.5 to 30.5–35.5	0.039 ± 0.056	0.038 ± 0.067	0.039 ± 0.054
15.5–20.5 to 35.5–40.5	0.062 ± 0.069	0.096 ± 0.083	0.038 ± 0.050
15.5–20.5 to 40.5–45.5	0.040 ± 0.059	0.063 ± 0.077	0.023 ± 0.041
15.5–20.5 to 45.5–50.5	0.071 ± 0.068	0.063 ± 0.077	0.077 ± 0.066
20.5–25.5 to 20.5–25.5	0.008 ± 0.000	0.008 ± 0.000	0.008 ± 0.000
20.5–25.5 to 25.5–30.5	0.093 ± 0.066	0.126 ± 0.069	0.070 ± 0.058
20.5–25.5 to 30.5–35.5	0.036 ± 0.051	0.054 ± 0.064	0.023 ± 0.040
20.5–25.5 to 35.5–40.5	0.089 ± 0.062	0.093 ± 0.079	0.086 ± 0.054
20.5–25.5 to 40.5–45.5	0.100 ± 0.058	0.116 ± 0.064	0.088 ± 0.054
20.5–25.5 to 45.5–50.5	0.049 ± 0.063	0.063 ± 0.077	0.040 ± 0.054
25.5–30.5 to 25.5–30.5	0.008 ± 0.000	0.008 ± 0.000	0.008 ± 0.000
25.5–30.5 to 30.5–35.5	0.066 ± 0.063	0.041 ± 0.073	0.084 ± 0.052
25.5–30.5 to 35.5–40.5	0.105 ± 0.063	0.126 ± 0.069	0.090 ± 0.059
25.5–30.5 to 40.5–45.5	0.062 ± 0.068	0.070 ± 0.085	0.057 ± 0.061
25.5–30.5 to 45.5–50.5	0.067 ± 0.064	0.063 ± 0.077	0.070 ± 0.059
30.5–35.5 to 30.5–35.5	0.008 ± 0.000	0.008 ± 0.000	0.008 ± 0.000
30.5–35.5 to 35.5–40.5	0.027 ± 0.043	0.031 ± 0.051	0.023 ± 0.040
30.5–35.5 to 40.5–45.5	0.079 ± 0.065	0.093 ± 0.079	0.068 ± 0.057
30.5–35.5 to 45.5–50.5	0.018 ± 0.033	0.031 ± 0.052	0.008 ± 0.000
35.5–40.5 to 35.5–40.5	0.008 ± 0.000	0.008 ± 0.000	0.008 ± 0.000
35.5–40.5 to 40.5–45.5	0.045 ± 0.054	0.031 ± 0.052	0.054 ± 0.058
35.5–40.5 to 45.5–50.5	0.062 ± 0.069	0.073 ± 0.089	0.054 ± 0.058
40.5–45.5 to 40.5–45.5	0.008 ± 0.000	0.008 ± 0.000	0.008 ± 0.000
40.5–45.5 to 45.5–50.5	0.067 ± 0.064	0.087 ± 0.075	0.053 ± 0.056
45.5–50.5 to 45.5–50.5	0.008 ± 0.000	0.008 ± 0.000	0.008 ± 0.000

### Resolved connector hubs for expressive language

**FIG. 3.**
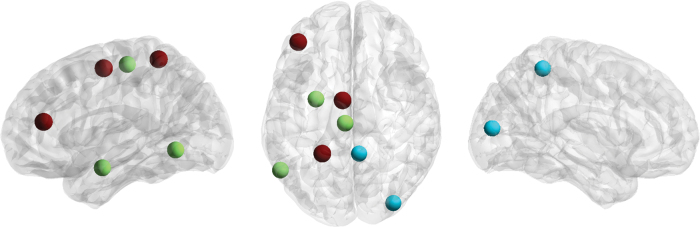
Connector hubs resolved. These include left superior frontal gyrus, left ventral middle frontal gyrus, left paracentral lobule, left ventrolateral inferior temporal gyrus, left postcentral superior parietal lobule, left lateral amygdala, right medial precuneus, and right middle occipital gyrus. Effect size is indicated by region color (blue to red). Color images are available online.

Eight connector hubs were resolved in the present analysis, including left superior frontal gyrus, left ventral middle frontal gyrus, left paracentral lobule, left ventrolateral inferior temporal gyrus, left postcentral superior parietal lobule, left lateral amygdala, right medial precuneus, and right middle occipital gyrus (Fig. 3).

### Comparison of multilayer versatility with DCI

**FIG. 4.**
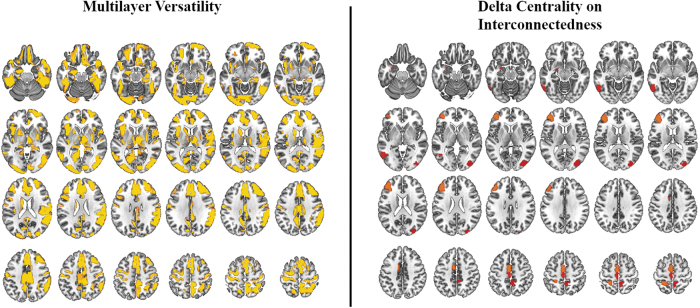
Group-level results of multilayer versatility compared with DCI. While both methods resolve regions implicated in expressive language, DCI seems to yield results with greater focality. Effect size indicated by color scale (red to yellow). DCI, delta centrality on interconnectedness. Color images are available online.

To determine the information gained by reranking the hubs obtained from multilayer versatility, statistical maps (using the same permutation testing used to obtain DCI maps) of versatility were visually compared with DCI results. Both methods resolved regions thought to be involved in expressive language. However, DCI had greater focality in resolving regions particularly linked to expressive language. Maps of nodes with significant multilayer versatility were much more diffuse and encompassed regions thought to be involved in a variety of cognitive functions (Fig. 4).

### Utility of structural constraint

**FIG. 5.**
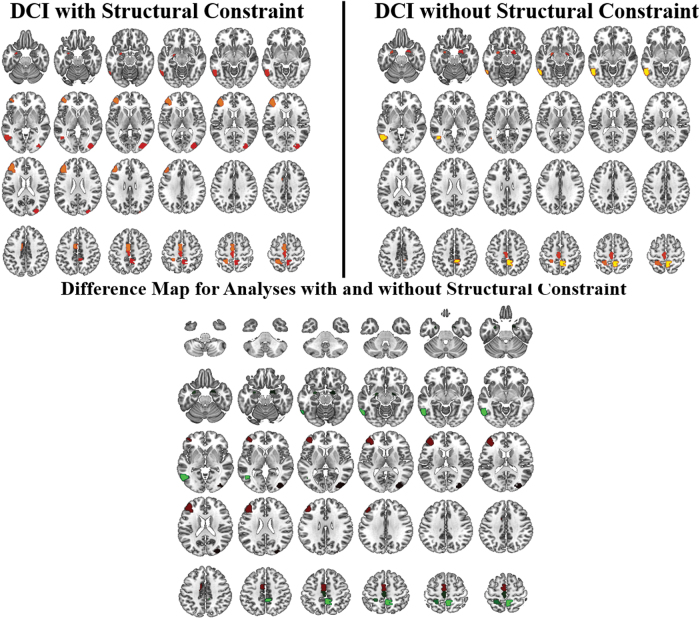
Group-level results comparing DCI without and with the structural constraint. With the structural constraint, results show a hub in the left middle frontal gyrus, which is not present when the analysis is performed without the structural constraint. The results with the structural constraint are also more left lateralized. Color images are available online.

One of the important innovations in the current study is the inclusion of the structural constraint to limit connections to only those that are biologically plausible, as determined by diffusion tractography. To show the utility of this approach, DCI results obtained from analysis both with and without the structural constraint were visually compared. Both methods resolved regions that have been implicated in language functioning. However, results without the constraint were more bilateral. Structurally constrained results showed greater reliability, resolving left paracentral regions and left middle frontal gyrus (Fig. 5).

### Individual-level results

**FIG. 6.**
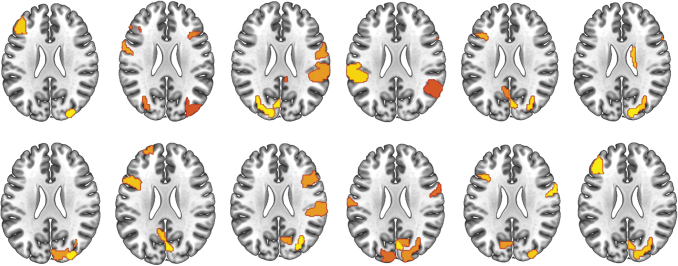
Individual maps (12 subjects) of regions resolved using the DCI approach. All maps are uncorrected at *p* < 0.001. DCI reliably resolves regions thought to support expressive language in 8 out of 12 subjects. Color images are available online.

Each individual statistic map was thresholded at an uncorrected *p* < 0.001 to assess the sensitivity of DCI at the subject level. DCI consistently resolved expressive language regions in all 12 subjects (Fig. 6). These results survived stricter thresholds and only regions thought to be directly involved in expressive language were resolved in 8 out 12 of the participants at the strictest threshold to still have results. The most consistent regions at the strictest threshold were the left middle frontal gyrus and left parietal lobule resolved in the group analysis. In the other four participants, the most robust result was dominated by parietal regions or right hemisphere homologues of the regions resolved in the other participants.

## Discussion

Overall, the present study showed the utility of connector hub analysis to delineate regions previously associated with expressive language functioning. This analysis has three key innovations compared with previous work: (1) using a data-driven multilayer framework utilizing MEG cross-frequency connectivity data to take advantage of rich, frequency-specific connectivity patterns, (2) using a structural constraint derived from state-of-the-art diffusion tractography to restrict functional connections to those that are biologically plausible, and (3) using a novel metric (DCI) that does not necessitate community detection to determine connector hubs in the multilayer network. This pipeline is fully data-driven and adds to the interpretation of how the resolved connector hubs contribute to expressive language, that is, these regions are necessarily important for interfrequency communication.

Two notable aspects of the results are the contrasts between hubs resolved between multilayer versatility and DCI and between analyses with and without the structural constraint. Multilayer versatility has previously been used successfully with resting-state fMRI data to distinguish schizophrenic patients from healthy controls (De Domenico et al., 2016). However, when applied to task-based data, results from the current study seem to show that versatility is biased toward resolving *central hubs*, often called the “rich club” of the brain (van den Heuvel and Sporns, 2011). These nodes are generally thought to be domain general and nonspecific for a given task. Reranking nodes by DCI resolves d*omain-specific* nodes that facilitate task-specific functioning between different brain regions.

Results were also improved by adding a structural constraint to the network. Importantly, this allows only the biologically plausible connections to be included in analyses. Previous studies have shown, in a multilayer framework, that functional connectivity is nontrivially constrained by structural connectivity (Battiston et al., 2017). Without the structural constraint, there is greater chance of noise and spurious correlation between two regions that are not directly connected. This constraint is crucial for reducing false connections and improving specificity.

Different areas of the brain have been shown to engage at different frequencies (Niedermeyer, 1999). This allows regionally variant natural frequencies, which is the ensuing frequency of oscillations after perturbation, for optimal flexibility. Researchers have suggested that connector hubs are optimally placed at locations where several cognitive systems overlap and may coordinate/mediate changes in connectivity between other pairs of nodes (Bertolero et al., 2018; Cole et al., 2013; Gratton et al., 2018; Spadone et al., 2015).

Studies of connector hub microstructure have revealed cytoarchitectural network features correlated with macroscale white matter connectivity features that distinguish connector hubs from other regions. For example, connector hub centrality was associated with more complexity of layer III pyramidal neurons, including more elaborate dendritic branching, larger soma, higher total spine count, more regional variation in total cell and neuron count, greater metabolic demand, and larger dendritic trees, allowing for a longer and more diverse connectivity profile (Scholtens et al., 2014). These properties may be especially important for expressive language, which involves coordination of several discrete processes (phonemic decoding, comprehension, semantics, etc.) Connector hubs derived from the current framework are likely crucial for facilitating connectivity between regions operating at different frequencies.

Delineation of connector hubs at the individual level also showed promise for clinical applications. For example, noninvasively mapping language function in the brain in patients undergoing cortical resection for intractable epilepsy is a significant challenge. While noninvasive functional imagings, such as fMRI and MEG, are adequate at lateralizing language function, their lack of concordance with “gold standard” invasive mapping limits their clinical utility (Binder, 2010; Rodin et al., 2013). One possible reason for this is that traditional functional imaging analyses cannot distinguish regions that are task essential versus those that are task correlated. Since our results consistently resolved regions thought to be crucial for language function, our pipeline may provide an alternative analysis to improve the concordance between noninvasive imaging and invasive mapping.

One limitation of this study is the quality of the diffusion data. The diffusion acquisitions were not prioritized in this study; instead, a brief, low-density, single-shell stock sequence was used, inherently limiting the ability to resolve crossing fibers and control for partial volume effects. Ongoing studies are using advanced diffusion imaging acquisition schemes and analyses to more accurately characterize the structural connectivity profile for each subject (Tournier et al., 2004; Tuch, 2004; Wedeen et al., 2005; Yeh et al., 2010). Another limitation was the use of a covert task during scanning that made it difficult to assess if and when the participant was successfully completing the task. Covert responses were obtained to limit artifact due to head movement. Wearable MEG systems are being developed, which would permit movement, allowing overt responses without signal corruption (Boto et al., 2017).

Future studies will aim to investigate the nature of connector hubs in other cognitive domains. It is necessary to determine if these connector hubs are unique to expressive language or if they are involved in other cognitive domains as well. The present findings can be incorporated in computational models to better simulate brain function and psychological findings. While neuroimaging data is expensive and labor intensive, computational tools and generative models can serve as investigative methods between studies and are crucial to the advancement of theories that lead to new experimentation (Betzel and Bassett, 2017).

Current computational models, specifically connectionist models (i.e., neural networks), have shown advantages in learning accuracy and vast gains in learning efficiency by incorporating neurophysiological properties (Guerguiev et al., 2017). Restructuring networks to more closely resemble empirical brain network topology may further improve these models and provide insight to learning in the brain. Finally, data are currently being collected to assess the viability of using this pipeline as a clinical mapping tool. We aim to validate the current pipeline with data from electrocortical stimulation mapping and semi-invasive transcortical magnetic stimulation studies.

## Conclusion

This framework reforms the idea of specialized functions for specific areas in the brain. For example, instead of a specific region being the “seat” of expressive language, the present study suggests that crucial hubs may be conduits for interfrequency communication between action and perception systems that are crucial for typical functioning (Pulvermüller, 2018). Importance of a particular region is not due to an instantiated function, but to its connectivity profile and the communication it affords.

## Supplementary Material

Supplemental data

Supplemental data

Supplemental data

## Appendix

### Appendix A1. Multilayer Versatility

Multilayer PageRank versatility has been introduced as a generalization of the classical PageRank centrality for single-layer networks. Classically, the PageRank centrality quantifies the probability that a random walker visits a given node by: (1) traversing node links and (2) teleporting through nodes at random. In the more general case of multilayer networks, random walk dynamics is governed by a transition tensor encoding the probability of walking through a link $\left(i,\alpha ,j,\beta \right)$ as

$$R_{j\beta }^{i\alpha }=rT_{j\beta }^{i\alpha }+\frac{\left(1-r\right)}{NL}u_{j\beta }^{i\alpha }$$

where *r* is a constant expressing the probability for the random walker to teleport. As in the algorithm developed by Brin and Page for Google Search, this parameter is fixed to 0.85 (Page et al., 1999). Here, $T_{j\beta }^{i\alpha }$ is the transition tensor whose entries are zero if there is no link between node *i* in layer α and node *j* in layer β and, otherwise, it is equal to the inverse of the number of (intra- and inter-) links incident to node *i* in layer α. Note that $u_{j\beta }^{i\alpha }=1$ for any choice of the indices.

Denoting by $\Omega_{i\alpha }$ the eigentensor of $R_{j\beta }^{i\alpha }$, calculated by flattening the tensor into its supra-adjacency representation (De Domenico et al., 2013; Gómez et al., 2013), the PageRank versatility $\omega_{i}$ of a node is obtained by summing across layers the score of its replicas as

where *L* is the number of layers. It is worth remarking that, as for its single-layer counterpart, the PageRank versatility can be calculated for all types of networks, including directed, weighted, and disconnected multilayer networks.
