# Supplementary figures and images for: Multilayer Connector Hub Mapping Reveals Key Brain Regions Supporting Expressive Language
Source: Brain Connect. 2021 Feb 12;11(1):45–55. doi: 10.1089/brain.2020.0776 (PMC7891212; doi:10.1089/brain.2020.0776)

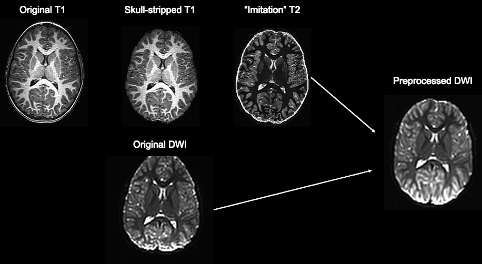

Supplement: Supplemental data [file Supp_FigS1.tif]

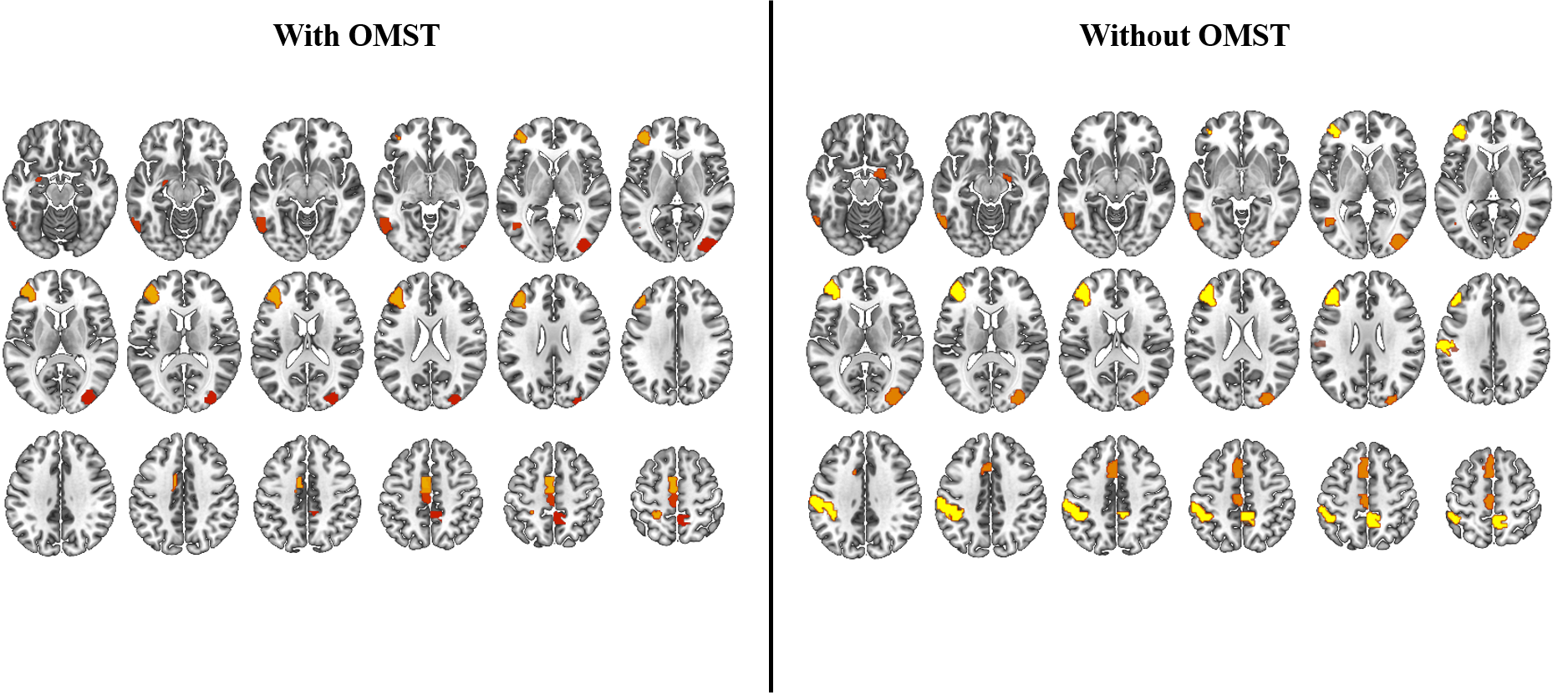

Supplement: Supplemental data [file Supp_FigS2.tif]

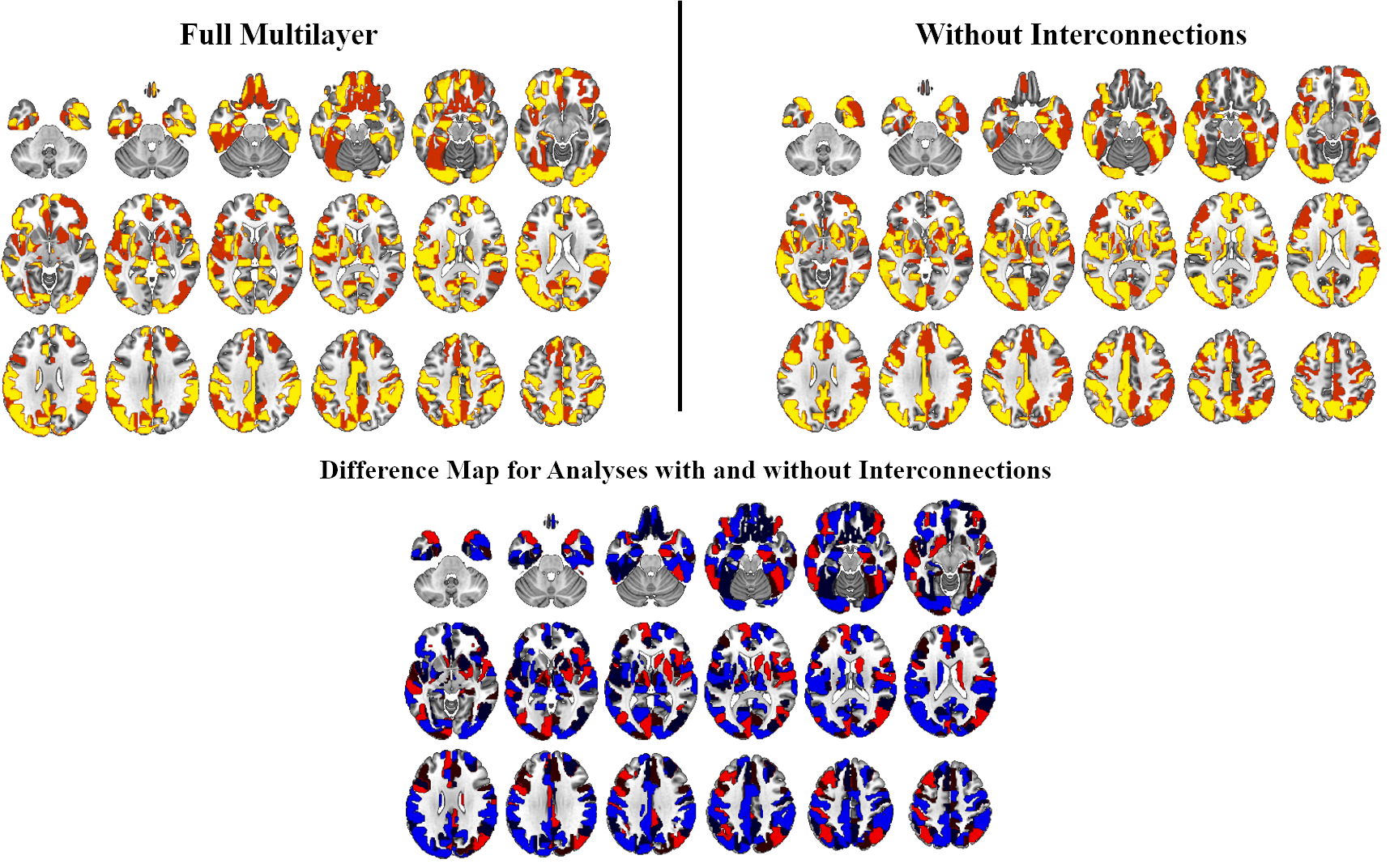

Supplement: Supplemental data [file Supp_FigS3.tif]
